# Differential interaction between DARC and SDF-1 on erythrocytes and their precursors

**DOI:** 10.1038/s41598-019-52186-6

**Published:** 2019-11-07

**Authors:** T. R. L. Klei, F. Aglialoro, F. P. J. Mul, S. Tol, P. C. Ligthart, I. M. Seignette, J. Geissler, E. van den Akker, R. van Bruggen

**Affiliations:** 1 Department of Blood Cell Research, Sanquin Research and Landsteiner Laboratory, Amsterdam UMC, Amsterdam, The Netherlands; 2 Department of Hematopoiesis, Sanquin Research and Landsteiner Laboratory, Amsterdam, Amsterdam The Netherlands; 3 Research facility, Sanquin Research and Landsteiner Laboratory, Amsterdam UMC, Amsterdam, The Netherlands; 4 Erythrocyte Serology, Sanquin, Amsterdam, The Netherlands

**Keywords:** Chemokines, Molecular biology

## Abstract

The Duffy Antigen Receptor for Chemokines (DARC) is expressed on erythrocytes and on endothelium of postcapillary venules and splenic sinusoids. Absence of DARC on erythrocytes, but not on endothelium, is referred to as the Duffy negative phenotype and is associated with neutropenia. Here we provide evidence that stromal cell-derived factor 1 (SDF-1), the chemokine that restricts neutrophil precursors to the bone marrow, binds to erythrocyte progenitors in a DARC-dependent manner. Furthermore, we show that SDF-1 binding to DARC is dependent on the conformation of DARC, which gradually changes during erythroid development, resulting in the absence of SDF-1 binding to mature erythrocytes. However, SDF-1 binding to erythrocytes was found to be inducible by pre-treating erythrocytes with IL-8 or with antibodies recognizing specific epitopes on DARC. Taken together, these novel findings identify DARC on erythrocyte precursors as a receptor for SDF-1, which may be of interest in beginning to understand the development of neutropenia in situations where DARC expression is limited.

## Introduction

DARC is an atypical chemokine receptor that, although structurally similar to G-protein coupled receptors, does not signal due to lack of the typical DRYLAIV G-protein-coupling sequence^1^. Although DARC on endothelial cells mediates internalization and transport of chemokines across endothelial barriers^2^, thereby possibly affecting leukocyte migration, the function of DARC on erythrocytes is not entirely clear. As DARC mainly interacts with inflammatory chemokines^3,4^ it is believed that erythrocytes, during infection, act as a sink for circulating pro-inflammatory chemokines^4,5^. An important clue to DARC function is the strong correlation between the erythroid DARC negative (Fy^−/−^) phenotype and neutropenia^6,7^. Moreover, a recent study in a murine model showed that absence of DARC on nucleated erythroid cells alters transcriptional programming of hematopoietic stem cells. In addition, lack of DARC expression on erythrocyte precursors, in combination with DARC expression on endothelial cells in the spleen, a situation which is comparable to the Fy^−/−^ phenotype in the human system, resulted in neutropenia^8^. However, the exact mechanism by which DARC influences the hematopoietic niche and the localization of neutrophils is still unclear. We hypothesized that stromal cell-derived factor 1 (SDF-1), the key chemokine that retains neutrophils and haematopoietic stem cells in the bone marrow during steady state, binds to erythrocyte precursors in a DARC-dependent manner,^9,10^. However, SDF-1 has been reported not to interact with DARC on mature erythrocytes^3,5,11^. As we recently provided evidence that DARC has different binding characteristics on reticulocytes compared to mature erythrocytes^12^, we determined SDF-1 binding to DARC on different stages of erythroid maturation to erythrocytes.

## Materials and Methods

### Blood and bone marrow samples, antibodies and materials

Blood studies were approved by the Medical Ethical Committee of Sanquin Research and performed in accordance with the 2013 Declaration of Helsinki. Bone marrow aspirates were obtained after informed consent from patients undergoing cardiac surgery according to the protocol for collecting bone marrow for research purposes as previously described^13^.

### Western blot, imaging, erythroblast culturing

Reticulocytes were sorted based on CD235a and CD71 expression by the AriaIII Cell sorter (BD Biosciences, Franklin Lakes, USA) lysed in Laemmli sample buffer and subjected to SDS-PAGE (Whatman, Little Chalfont, UK). Erythroblasts and reticulocytes of mixed stages were cultured as described perviously^14–16^. Imaging flow cytometry was performed on the Amnis Imaging Flow Cytometer (Millipore, Burlington, USA).

### Flow cytometry staining procedures

For every flow cytometric experiment where we determine SDF-1 binding to erythrocytes and its precursors we used biotinylated SDF-1 antibody listed in supplementary table 1 followed by streptavidin-647 conjugation. Afterwards, we took along nuclear staining (Hoechst), anti-transferrin receptor (anti-CD71-FITC) and glycophorin-A staining (anti-CD235a-PE) to distinguish between the various stages of erythroid development. In case we had to perform additional stainings, such as in the case of determining Fy epitope exposure (e.g. Fy^a^, Fy^b^, Fy^3^ or Fy^6^) on SDF-interacting and non-interacting reticulocytes, we switched the order to ensure antigen-specific staining. In short, we first stained for Fy epitopes followed by either secondary anti-human-405 (for Fy^a^ and Fy^b^) or anti-mouse-405 (for Fy^3^ or Fy^6^) after which we stained for SDF followed by streptavidin-647 conjugation, after which we again took along anti-CD71-FITC and anti-235a-PE). To further ensure antigen-specific staining we took along the appropriate IgG isotype controls. Exogenous addition of SDF-1 to erythroid cells was performed at 37 °C for 30 minutes whereas staining was performed at 4 °C. Flow-cytometry analysis was performed on LSRII + HTS and data were analysed by FACSDiva software (BD Biosciences, Franklin Lakes, USA).

## Results and Discussion

### Reticulocytes bind SDF-1

**Figure 1:**
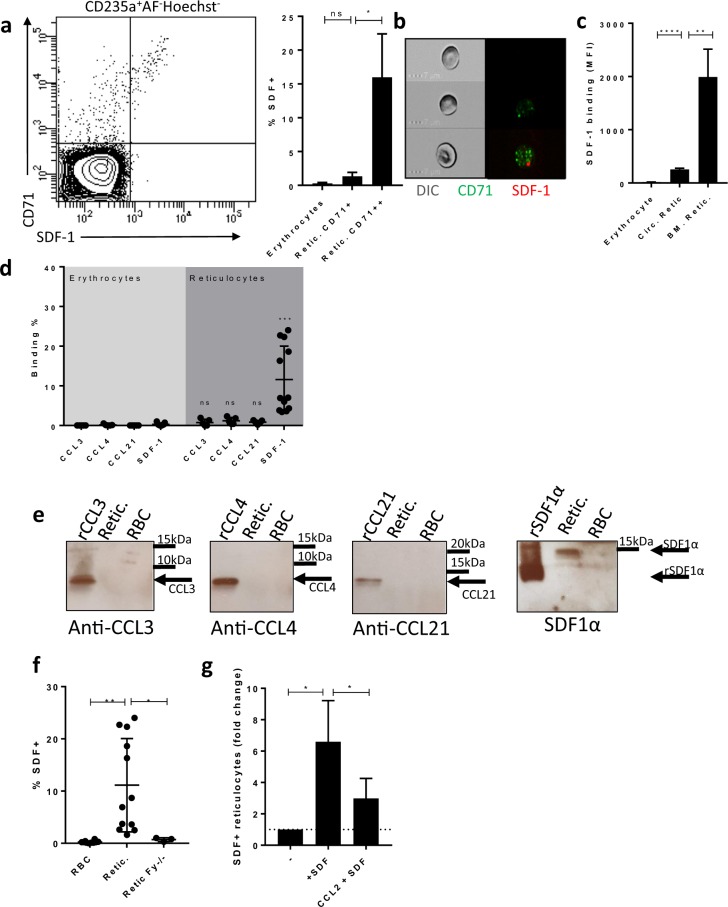
Reticulocytes and their progenitors bind SDF-1. **(a)** Flow-cytometry gating strategy to determine the number of erythrocytes that bind SDF-1 (% SDF+) as well as the relative amount of SDF-1 that is bound (mean fluorescence intensity) on erythrocytes and reticulocytes from circulation and bone marrow. Erythrocytes are defined as autofluorescent, nucleus and transferrin receptor negative (AF^−^, hoechst^−^CD71^−^) and glycophorin-A positive (CD235a^+^) whereas reticulocytes are defined as AF^−^Hoechst^−^CD235a^+^CD71^+^. (n = 3–6, Paired T-test; ns, not significant; *P < 0.05). **(b)** Imagestream micrograph of freshly drawn erythrocytes and reticulocytes showing that SDF-1 binding to CD71^++^ reticulocytes is punctate. **(c)** Quantification of erythrocytes, circulating (Circ) and bone marrow (BM) reticulocytes binding to SDF-1. **(d)** Quantification of the percentage of erythrocytes and reticulocytes interacting with SDF-1, CCL3, CCL4 and CCL21 (n = 6–12, Paired T-test; ***P < 0.001). **(e)** Red blood cells (RBC) were isolated from circulation as described in the methods section. Reticulocytes (Retic.) were MACS isolated using CD71 antibody. Erythrocytes, reticulocytes and purified recombinant chemokines (rCCL3; 7.8 kDa, rCCL4; 7.6 kDa, rCCL21; 12.2 kDa and rSDF-1; 8 kDa) were subjected to SDS page and western blot. In line with flow cytometry data, CCL3, CCL4 and CCL21 were not detected on both erythrocytes as well as on reticulocytes. SDF-1 (~10.6 kDa) however was detected only on reticulocytes and not on erythrocytes. The molecular weight difference between recombinant and reticulocyte endogenous SDF-1 may result from post-translation modification or dimerization of SDF-1. **(f)** Quantification of SDF-1 binding to erythrocytes and reticulocytes from DARC negative and positive individuals (n = 3–12, unpaired T-test; *P < 0.05;**P < 0.01). **(g)** Effect of exogenous addition of SDF-1 to erythrocytes and reticulocytes in the absence and presence of equimolar levels of CCL2 (n = 5, paired T-test; *P < 0.05).

To test if DARC differentially binds SDF-1 during erythrocyte maturation we first assessed membrane-bound SDF-1 on erythrocytes and reticulocytes. We found that the most immature CD71^high^ reticulocytes in the circulation, and to a lesser extent CD71^low^ reticulocytes, but not erythrocytes, bind SDF-1 (Fig. 1a). These data were further supported by imagestream analysis (Fig. 1b). We found that SDF-1 binding to bone marrow reticulocytes (the most immature reticulocytes) was even higher in comparison to reticulocytes and erythrocytes from peripheral blood (Fig. 1c). In contrast, various other chemokines that have previously been described, similar to SDF-1, not to interact with erythrocytes^5^, did not bind reticulocytes (Fig. 1d). In agreement with the flow cytometry data, western blot analysis showed that CCL3, CCL4 and CCL21, in contrast to SDF-1, were not detected on the membranes of both erythrocytes and reticulocytes (Fig. 1e). Note that recombinant SDF-1 runs at a lower molecular weight as compared to endogenous SDF-1 (See Supplemental Table 1 for specifics). Importantly, we found that SDF-1 did not bind to reticulocytes isolated from Fy^−/−^ individuals (Fig. 1f), suggesting that SDF-1 binding to reticulocytes depends on expression of DARC. In addition and in support of this hypothesis, CCL2, a known DARC-binding chemokine^17^, inhibits SDF-1 binding to DARC^+^ reticulocytes (Fig. 1g). Thus we suggest that SDF-1 binds to DARC on immature CD71^+^ reticulocytes and this interaction is lost upon reticulocyte maturation to erythrocytes.

### Erythrocyte precursors bind SDF-1

**Figure 2:**
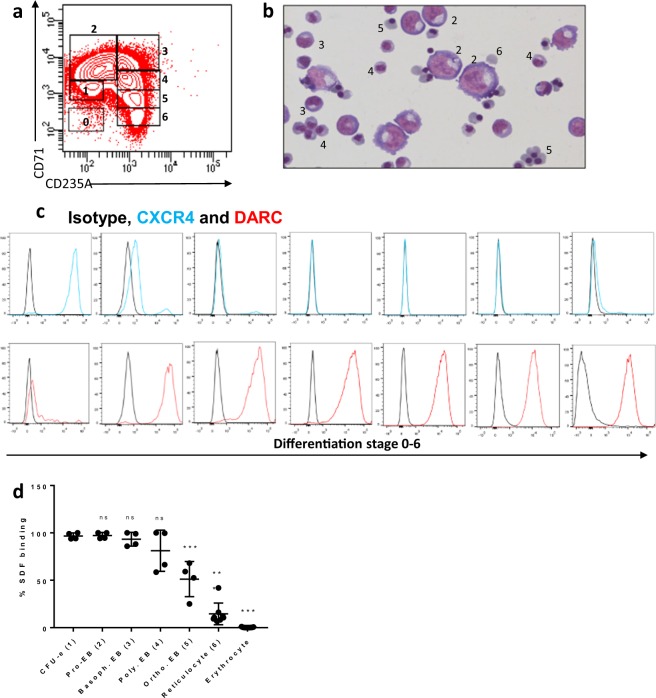
Erythroid progenitors bind SDF-1. **(a)** Flow cytometric dot-plot used to define various stages of cultured erythroblasts and reticulocytes based on CD71 and CD235a expression. Populations are designated as follows; 0: Peripheral Blood Mononuclear Cells (PBMCs), 1: CFU-E, 2: Pro-erythroblast, 3: Basophilic erythroblast, 4: Polychromatic erythroblast, 5: Orthochromatic erythroblast, 6: late orthochromatic erythroblasts/reticulocytes. **(b)** May-Grünwald/Giemsa staining of the erythroblast culture that was used to assess SDF-1 binding capacity. Asynchronous erythroblast differentiation cultures were used in order to assess SDF-1 binding capacity during differentiation (2: Pro-erythroblast, 3: Basophilic erythroblast, 4: Polychromatic erythroblast, 5: Orthochromatic erythroblast, 6: reticulocyte) **(c)** Representative histograms of Fy^a^ epitope of DARC and CXCR4 expression by erythroblasts during differentiation **(d)** Quantification of SDF-1 binding by the various stages (0–6 corresponding to figure E) of cultured erythrocyte progenitors and circulation-derived erythrocytes. 1 µg/ml SDF-1 was exogenously added to cultured erythroblasts. (1-way Anova *P < 0.05; **P < 0.01; ***P < 0.001).

DARC is expressed in erythroid precursors^18^. As reticulocytes bind SDF-1 in a DARC-dependent manner this may suggest that erythrocyte precursors may also bind SDF-1 through DARC. *In vitro* cultured erythroid precursors can be ranked from early pro-erythroblasts to late enucleated reticulocytes. We defined several erythroblast populations based on CD71 and CD235a expression^19^ (Fig. 2a,b). The bona fide receptor of SDF-1 is CXCR4, which expression was quickly downregulated at the onset of erythroblast differentiation (Fig. 2a,c). DARC was already expressed on erythroblasts and expression was maintained during differentiation (Fig. 2c). Next we assessed SDF-1 binding dynamics during erythroblast differentiation. Strikingly, we found that almost all erythrocyte precursors were capable of binding SDF-1. This was found to gradually decrease during their maturation into reticulocytes (Fig. 2d, Suppl. Fig. 1a). These results suggest that SDF-1 binding to erythrocyte precursors is dependent on the erythroid maturation stage and that CXCR4 is not involved in this as CXCR4 expression is quickly downregulated at the onset of differentiation.

### DARC epitope exposure is influenced upon SDF-1 binding

**Figure 3:**
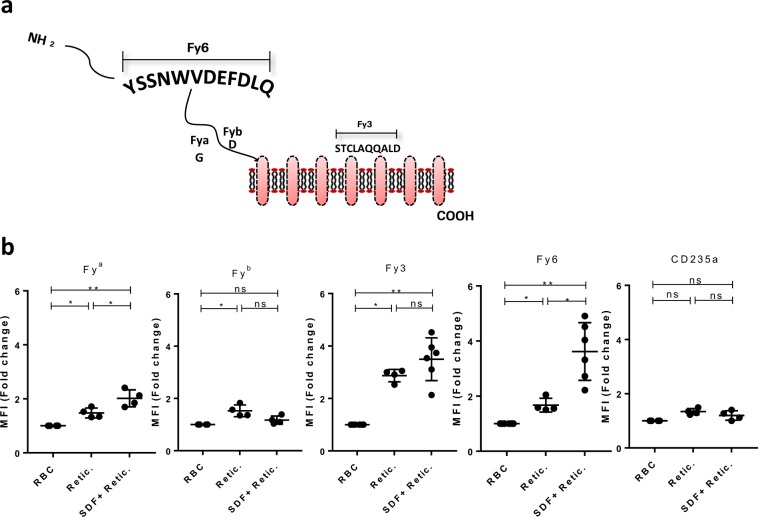
Differential Fy epitope exposure on SDF-1-interacting reticulocytes. **(a)** Schematic representation of DARC membrane protein, including Fy^A^, Fy^B^, Fy^3^, Fy^6^ epitopes. **(b)** Mean fluorescence intensity (MFI) of DARC epitope Fy^6^ Fy^a^, Fy^b^, Fy^3^ and Glycophorin-A, an erythroid specific marker, used as a control (CD235a), on erythrocytes (RBC), reticulocytes (retic.) and SDF-1 positive reticulocytes (SDF-1 + Retic.), presented in fold change and normalized to erythrocytes. (Paired T-test, n = 4–5, ns: not significant; *P < 0.05; **P < 0.01; ***P < 0.001).

The region between the N-terminal domain that carries the Fy^6^ epitope and the fourth extracellular domain within DARC, is required to switch to an active chemokine‐binding pocket^20^ (Fig. 3a). In addition, we previously reported increased accessibility of the DARC Fy6 epitope within immature reticulocytes compared to erythrocytes^12^. Therefore we assessed if the accessibility of specific epitopes within DARC, and in particular epitope Fy6, is increased on SDF-1-interacting reticulocytes from the circulation. We found an increased association of Fy^6^ epitope recognizing antibodies on SDF-interacting reticulocytes, as compared to those that did not contain membrane bound SDF-1 (Fig. 3b). To a significantly lesser extent this was also observed for Fy^a^. SDF-1 binding did not affect the association of antibodies to Fy^b^, Fy^3^ or the control CD235a. This suggests that increased exposure of the Fy^6^ epitope within DARC may be required for SDF-1 binding, similar to what is observed in binding of *Plasmodium vivax* to DARC on reticulocytes^12^.

### SDF-1 binding to DARC on erythrocytes and reticulocytes is inducible

**Figure 4:**
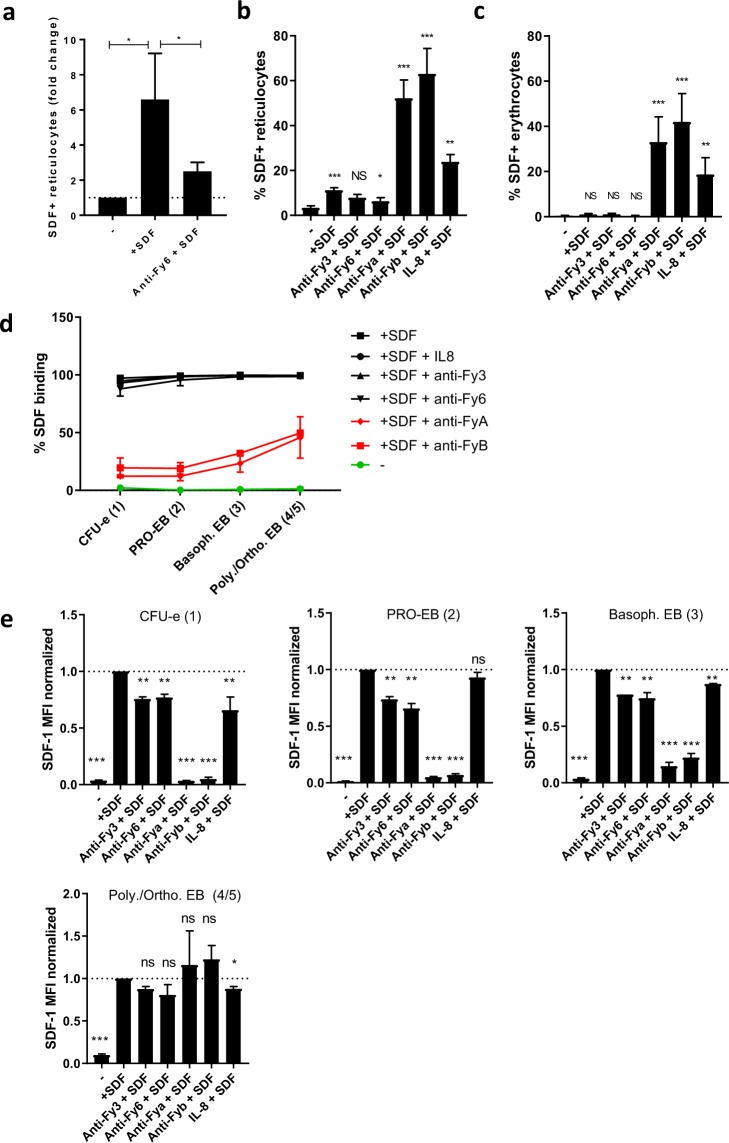
SDF-1 binding capacity in erythrocytes can be rescued. **(a)** Effect of exogenous addition of SDF-1 to reticulocytes in the absence and presence of anti-Fy^6^ (n = 5, one-way anova *P < 0.05). **(b**–**e)** Effect of exogenous addition of SDF-1 to erythrocytes, reticulocytes and erythroblasts of various stages (as determined in Fig. 2a) in the absence and presence of anti-Fy^3^, anti-Fy^6^, anti-Fy^a^, anti-Fy^b^ or IL-8. (n = 3–5, one-way anova *P < 0.05).

Next we investigated if an antibody specific to the Fy6-epitope would interfere with SDF-1 binding. Indeed, blocking the Fy^6^ epitope prior to exogenous addition of SDF-1 resulted in a significant reduction of SDF-1 binding (Fig. 4a). This finding suggests that exposure of the Fy6 epitope is altered on immature reticulocytes and may be required for SDF-1 binding. Unexpectedly, in contrast to decreased SDF-1 binding to reticulocytes due to blocking with anti-Fy^6^ antibody, both Fy^a^ and Fy^b^ antibody binding led to increased SDF-1 binding. In addition, pre-treatment with IL-8, a chemokine known to bind to DARC, also increased SDF-1 binding to reticulocytes (Fig. 4b). This finding suggests that the binding of an antibody or a chemokine can cause changes in the exposure of specific parts within DARC on reticulocytes, allowing the binding of SDF-1. This led us to hypothesize that these antibodies/chemokines may render erythrocytes that normally are not capable of binding SDF-1 permissive to binding SDF-1. Indeed, binding of Fy^a^ and Fy^b^ antibodies as well as IL-8 to DARC allowed SDF-1 to bind to erythrocytes (Fig. 4c). These findings underscore that the ability of DARC to bind SDF-1 is not indefinitely lost on erythrocytes and can be modulated by chemokines. Together, these results show that DARC affinity for SDF-1 changes during reticulocyte maturation into erythrocytes and strongly suggests that SDF-1 binding is dependent on DARC conformation^8,21^. Next to performing these competition experiments on erythrocytes and reticulocytes, we also assessed the ability of anti-Fy^3^, anti-Fy^6^ and IL-8 to interfere in the interaction between DARC and SDF-1 in erythroid precursors. We found that the percentage of erythroid precursors that bound SDF-1 upon co-incubation with anti-Fy^3^, anti-Fy^6^ and IL-8 was not affected (Fig. 4d). However, as observed in reticulocytes, a significant relative reduction of SDF-1 bound per erythroid precursor was observed, suggesting competition (Fig. 4e). In contrast to reticulocytes from circulation, anti-Fy^a^ and anti-Fy^b^ antibodies had a strong negative effect on SDF-1 binding to cultured erythroid precursors. This inhibiting effect became less apparent as erythroid precursors matured from CFU-e to orthochromatic/polychromatic erythroblasts, which is reflected both in the percentage of cells interacting with SDF1 (Fig. 4d) as well as the amount of SDF-1 bound (Fig. 4e), resembling more the circulating reticulocytes (Fig. 4b). These results underscore that SDF-1 binding to DARC, and the ability to induce or prevent this interaction from occurring, is dependent on the stage of maturation.

Taken together, we report the novel finding that, during maturation, erythroid precursors gradually down regulate the ability to bind SDF-1, and we propose that this binding is DARC-dependent. The antibody and chemokine binding assays indicate that, during terminal differentiation into the erythrocyte stage, the decreased binding of SDF-1 may be due to a conformational switch in DARC, which is a phenomenon that has been shown to account for the selective invasion of reticulocytes by *Plasmodium vivax*^12^. Recent research in a murine knockout model showed that DARC expression on erythrocyte progenitors is essential for the retention of immature neutrophils in the bone marrow and the spleen. Here we speculate that this retention could be dependent on SDF-1, the chemokine that is mostly known for restricting neutrophil precursors to the bone marrow. SDF-1 association to erythrocyte progenitors in a DARC-dependent manner may change the availability of SDF-1 within the bone marrow possibly affecting hematopoietic progenitor and effector cell (e.g. neutrophils) migration. This may begin to explain the neutropenia in Duffy negative individuals. It would be of great interest to further study the function of DARC in neutrophil localization and mobilization in and from the bone marrow.

## Supplementary information

Supplementary information
