# Supplementary material for: Differential interaction between DARC and SDF-1 on erythrocytes and their precursors
Source: Sci Rep. 2019 Nov 7;9:16245. doi: 10.1038/s41598-019-52186-6 (PMC6838059; doi:10.1038/s41598-019-52186-6)
Supplement: Supplementary file 1 — Supplementary information [file 41598_2019_52186_MOESM1_ESM.pdf]

Differential interaction between DARC and SDF-1 on erythrocytes and their precursors

T.R.L. Klei*, F. Aglialoro*, F.P.J. Mul, S. Tol, P.C. Ligthart, I.M. Seignette, J. Geissler, E. van den Akker and R. van Bruggen

Supplementary information

Supplementary Figure 1

(A) SDF-1 binding on in vitro cultured erythroid progenitors was assessed by image-stream analysis. Similar to SDF-1 distribution on circulation-derived reticulocytes, the distribution on cultured erythroblasts was found to be punctate. Erythroblasts were identified based on CD71 and CD235a expression as is also shown in figure 2a. SDF-1 binding is shown for erythroblasts that originate from population 2 as identified in figure 2a.

Supplementary figure 1

A

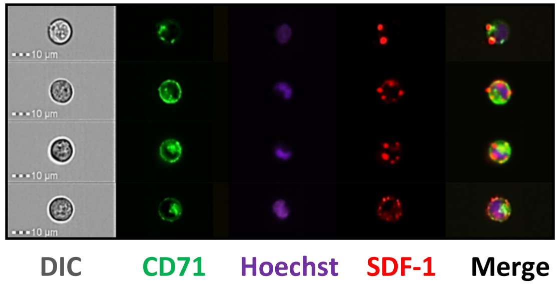

Supplementary table 1

Product	Source	Clone / Ref.	Staining Dilution	Blocking/binding dilution
Anti-CD235a-PE	Pelicluster Sanquin	M1732	1:50	
Anti-CD71-FITC	BD biosciences	OKT9	1:50	
Anti-CD71	OKT9 construct	OKT9	1:50	
Hoechst	Invitrogen		1:10000	
Anti-Fy[3]	Gift, Dr. Makoto Uchikawa, Japanese Red Cross Central Blood Center, Tokyo, Japan	MIMA-29	1:100	1:10
Anti-Fy[6]	Gift, Dr. Antoin Blancher, Université Paul Sabatier, Toulouse, France	CBC-173	1:100	1:10
Anti-Fy[a]	Pelicluster, Sanquin	FA215AX	1:50	1:10
Anti-Fy[b]	Pelicluster, Sanquin	FYB070ax	1:50	1:10
Anti-mouse AF488	Life technologies	A11001	1:200	
Anti-human AF488	Life technologies	a11013	1:200	
Anti-CXCL12-APC	R&D systems	79018	1:10	
Anti-CXCL12-Biotin	R&D systems	BAF310	1:2000	
Streptavidin-APC	Thermo Fisher	S32357	1:200	
Anti-Ve-Cadherin-AF647	Becton Dickinson	55-7H1	1:100	
Recombinant human CXCL12 (8 kDa)	Preprotech	300-28A		30nM
Biotinylated anti-human CCL3/4/21	Preprotech	500-P38GBT<br>500-P38BBT<br>500-P109BT	1:50	200µg/mL
Anti-CXCR4	R&D		10ul	
Anti-Flavocytochrome B558	MBL	7D5	1:200	
